# Synergetic Resonance Matching of a Microphone and a Photoacoustic Cell

**DOI:** 10.3390/s17040804

**Published:** 2017-04-08

**Authors:** Joo Yong Sim, Chang-Geun Ahn, Chul Huh, Kwang Hyo Chung, Eun-Ju Jeong, Bong Kyu Kim

**Affiliations:** Bio-Medical IT Convergence Research Department, Electronics and Telecommunications Research Institute, Daejeon 34129, Korea; jsim@etri.re.kr (J.Y.S.); cgahn@etri.re.kr (C.-G.A.); chuh@etri.re.kr (C.H.); hyo@etri.re.kr (K.H.C.); eunju@etri.re.kr (E.-J.J.)

**Keywords:** photoacoustic spectroscopy, mid-infrared spectroscopy, acoustic resonance

## Abstract

We propose an approach to match the resonant characteristics of a photoacoustic cell with that of a microphone in order to enhance the signal-to-noise ratio in the photoacoustic sensor system. The synergetic resonance matching of a photoacoustic cell and a microphone was achieved by observing that photoacoustic cell resonance is merged with microphone resonance, in addition to conducting numerical and analytical simulations. Using this approach, we show that the signal-to-noise ratio was increased 3.5-fold from the optimized to non-optimized cell in the photoacoustic spectroscopy system. The present work is expected to have a broad impact on a number of applications, from improving weak photoacoustic signals in photoacoustic spectroscopy to ameliorating various sensors that use acoustic resonant filters.

## 1. Introduction

Photoacoustic spectroscopy is a powerful analytical tool used to probe the information of chemical composites by measuring the optical absorption characteristics of the sample. In biological samples, mid-infrared photoacoustic spectroscopy has been found to be extremely useful for applications such as monitoring glucose non-invasively by probing the interstitial fluid through the skin [1,2,3,4]. To amplify the weak photoacoustic signal due to the impedance mismatch of the acoustic medium, sensitive photoacoustic detectors have used an acoustic resonator, referred to as a photoacoustic cell. Conventional photoacoustic spectroscopy uses a continuous-wave laser modulated by an optical chopper at a frequency typically lower than a few kHz, chosen in the flat band of the microphone spectrum [5,6,7]. According to the well-known Rosencwaig-Gersho model [8], optically thick samples, for instance, in the case of irradiating a mid-infrared (MIR) laser to biological tissues, have an inversely proportional relation between the thermal diffusion length and the modulation frequency. Therefore, the photoacoustic signal amplitude (*A_p_*) decreases with higher modulation frequency (*f*), *A_p_* ~ *f ^-3/2^* [9]. In contrast, in pulsed-laser photoacoustic spectroscopy, the thermal diffusion length is independent of the repetition frequency for constant pulse width, and thus it is necessary to modulate the laser at a higher frequency to achieve larger average power and lower acoustic noise in ultrasound. Researchers have discussed the theoretical behavior of acoustic resonant cells in the audible frequency range for conventional photoacoustic spectroscopy using a microphone [10,11,12]. In ultrasound, photoacoustic spectroscopy systems have been developed using a quartz tuning fork instead of a microphone [13,14,15,16]. Quartz-enhanced photoacoustic spectroscopy (QEPAS) is based on a resonantly operated acoustic sensor and acoustic cells optimized for the resonance of the quartz tuning fork [14,15,16]. However, the resonance of a conventional microphone with a resonance-matched photoacoustic cell has not yet been demonstrated. Such an approach is applicable to conventional photoacoustic spectroscopy, which widely uses commercial microphones as an enabling technology. For QEPAS, efforts have been made to decrease the resonance frequency and reduce the optical noise caused by light hitting the fork [17,18]. The use of a microphone resonance can provide an attractive alternative to address these challenges underlying QEPAS.

We previously reported an early version of our photoacoustic spectroscopy system with extensive resonance investigations of the same cell, regarding the dependency of the main cavity height. In this study, we report a sophisticated approach for optimization to find the dominant mode of resonance and match the resonances of a microphone and a photoacoustic cell. We observe that the resonance of the photoacoustic cell is clearly merged with and separated from the resonance of the microphone, and thereby aim to optimize our photoacoustic spectroscopy system through the alignment of the resonant characteristics. We determine the dominant mode of acoustic resonance using numerical and analytical simulations, and the results are shown to be consistent with the experimental results. This present work can provide guidance to optimize photoacoustic cells as well as generally influence various applications of photoacoustic sensors, such as non-invasive glucose monitoring [4,19], detecting trace gases [20,21], and quantifying other non-gaseous samples [22].

## 2. Materials and Methods

### 2.1. Measurements of Microphone Frequency Response

A SPM0404 microphone from Knowles Inc., whose working bandwidth includes an ultrasound range over 50 kHz, was used. The resonance characteristic of the microphone was determined by measuring the frequency response to the acoustic pulse signal from 1 kHz to 100 kHz. The acoustic pulse signal was generated by directly irradiating the microphone membrane with a mid-infrared laser beam guided through the microphone opening. An external-cavity quantum-cascade laser (Über Tuner 9, Daylight Solutions, Inc., San Diego, CA, USA) served as a laser source, which can tune the wavenumber from 950 cm^−1^ to 1250 cm^−1^ and modulate the repetition frequency ranging from 0.1 kHz to 100 kHz with a maximum pulse width of 500 ns. The maximum peak power of the laser was 150 mW and the laser beam size was 2 mm in diameter. The laser was set at a pulse duration of 500 ns and at a wavenumber of 1165 cm^−1^, which resulted in the largest photoacoustic signal among the available wavenumbers of the laser. The microphone signal was amplified by an instrumentation amplifier (INA103, Analog Devices, Inc., Norwood, MA, USA) and the amplitude and phase were picked up by a lock-in amplifier (SR830, Stanford Research Systems, Inc., Sunnyvale, CA, USA) with a time constant of 100 ms. These conditions were used for the rest of the photoacoustic signal characterization.

### 2.2. Measurements of Resonant Behavior of Photoacoustic Cells Combined with a Microphone

**Figure 1:**
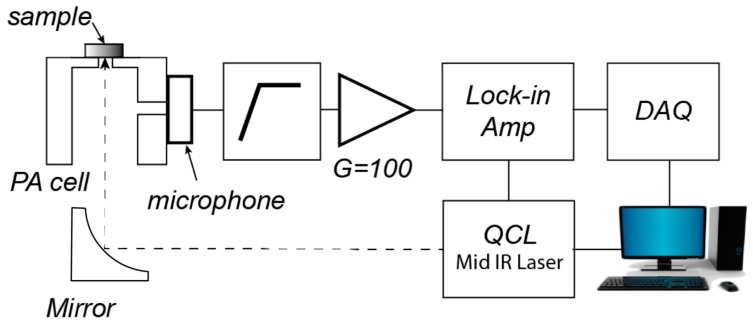
Block diagram of the apparatus for the photoacoustic spectroscopy measurement. The system consists of a mid-infrared (MIR) laser source, a laser controller, a data acquisition module, and a photoacoustic cell with an embedded microphone.

We built a photoacoustic spectroscopy system for measurements using an MIR laser, as described in Section 2.1. Figure 1 schematically illustrates the system. The system consisted of an MIR laser source, a laser controller, a data acquisition module, and a photoacoustic cell with an embedded microphone. The photoacoustic cell was composed of two perpendicularly intersecting cylindrical cavities made from stainless steel. It was previously demonstrated that this cell geometry, a so-called T-cell, allows independent optimization of geometric parameters to determine the signal strength [23]. The larger main cavity lets the laser beam enter one side and irradiates a sample located at the other end; the horizontally intersecting cavity, referred to as branch cavity, allows the attached microphone to probe the sound pressure generated by photoacoustic effects. A light-absorbing material, carbon black tape (NEM carbon black tape, Nissin Co. Ltd., Tokyo, Japan), served as a reference sample, which was placed on the opening of the main cavity. The laser beam was guided to the cell and focused on the sample through the opening site using a right-angle off-axis parabolic mirror (MPD019-M01, Thorlab, Inc., Newton, NJ, USA) with a 25.4 mm reflected focal length. The main cavity and branch cavity had the following default dimensions, respectively: 8.5 mm in diameter and 15 mm in height; 2 mm in diameter and 8 mm in length. The branch cavity was located 2 mm from the top seal of the main cavity to the center of the branch cavity. The main cavity had an opening at the sample side with a diameter of 2.5 mm. To determine the frequency response, we scanned the repetition frequency between 1 kHz to 80 kHz in a step of 0.1 kHz.

To optimize the resonant behavior of the cell, we manufactured many different cells for each altered geometry, while the architecture of the cavities remained the same. A microphone with a flat response could be used to measure the cell’s frequency response, but we directly measured the combined response of the acoustic cell and the microphone. If the size of the microphone used in the photoacoustic spectroscopy differs from a broadband microphone (e.g., a pressure field microphone with a bandwidth of up to 100 kHz from Brüel & Kjær Inc., Nærum, Denmark), a different mount should be used to locate and seal the two different microphones in the acoustic cell. A slight geometrical difference of the cells in close proximity to the microphone can lead to different acoustic behaviors of the acoustic cell. The frequency response of a microphone relies on the incident angle of an acoustic wave, and thus any varied reflection of the acoustic wave around the microphone may lead to inaccuracy of the measurements. Our method is based on the same microphone and mount for the microphone, and we can eliminate any possibility that different results occur when another reference microphone with a flat frequency response is used to optimize the acoustic resonant characteristics of the cell.

## 3. Results and Discussion

### 3.1. Frequency Response of the Microphone

**Figure 2:**
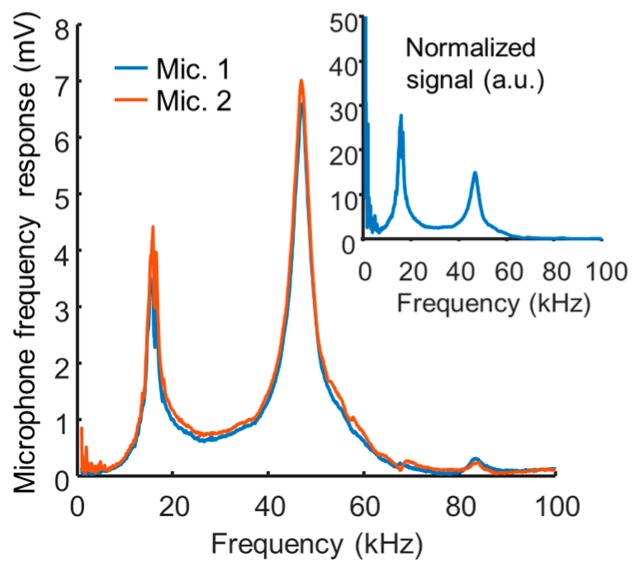
Frequency response of two microphones (Mic. 1 and Mic. 2) of the same type. (inset: normalized frequency response for the laser power, which increases proportionally to the repetition frequency).

Figure 2 shows the frequency response of the microphone, averaged over four repeated acquisitions. When the same microphone models were tested, they exhibited consistent frequency response curves (Mic. 1 and Mic. 2 in Figure 2). The inset of Figure 2 shows the signal normalized by the average laser power, which increases with the repetition rate. The average laser power increases with the repetition rate because we kept the pulse width constant, and thus a laser pulse with constant energy is given more frequently as the repetition rate becomes higher. The changing laser power was compensated by dividing the microphone signal by the repetition rate, where we assumed the average laser power increases linearly with the repetition rate. As a result, the microphone showed resonant peaks at 16 kHz and 47 kHz, and a highly sensitive range of 35 kHz–65 kHz in ultrasound. The Q-factors of the resonances were 6.1 at 16 kHz and 11.8 at 47 kHz. In our previous report [4], we generated an external soundwave by irradiating the laser beam onto a carbon black sample in front of the microphone’s opening and observed a similar frequency response to the frequency response of acoustic waves that were generated inside the microphone. The microphone membrane is typically designed to have higher resonance frequency than the working audible bandwidth, which is less than 20 kHz. Thus, the microphone resonance is the mechanical characteristics of the microphone membrane. The resonance of the microphone membrane relies not only on the diameter of the membrane (approximately 700 µm for the microphone we used) but on the thickness and material properties of the membrane. Here we optimize a photoacoustic cell for microphone resonance but, conversely, it would be possible to optimize a microphone for a photoacoustic cell.

### 3.2. Resonance Matching of Photoacoustic-Cell Resonace with Microphone Resonace

**Figure 3:**
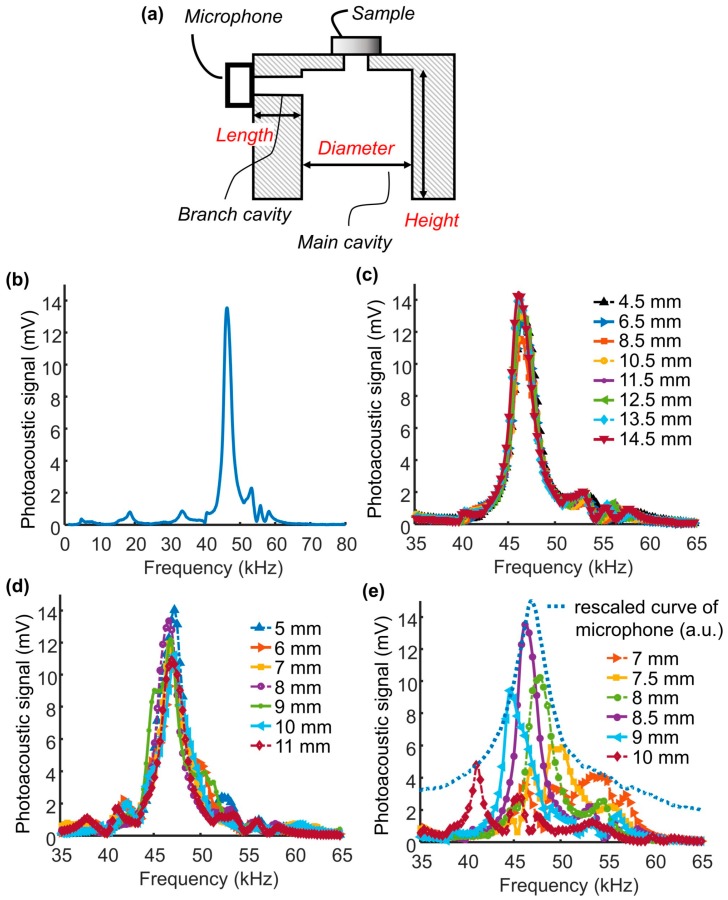
Frequency response of the photoacoustic cell dependent on the cell geometry. (**a**) Diagram of a photoacoustic cell consisting of two cavities, a branch cavity and a main cavity. (**b**) The frequency response of the default photoacoustic cell (8 mm length, 8.5 mm diameter, and 15 mm height). (**c**–**e**) Frequency responses of the photoacoustic cells measured when altering (**c**) the height of the main cavity, (**d**) the length of the branch cavity and (**e**) the diameter of the main cavity where the microphone frequency response was rescaled to show that it forms an envelope of the photoacoustic signal (Unit: arbitrary unit).

Next, we examined the resonance properties of the photoacoustic cell and its behavior combined with the microphone resonance. As a result, we obtained the largest signal at the frequency of 47 kHz, where the microphone was most sensitive (Figure 3b). Small peaks at 18.5 kHz and 33 kHz were observed near the first resonance peak of the microphone before the large peak of 47 kHz. The photoacoustic signal, depending on the modulation frequency, is a superposition of the characteristics of the photoacoustic cell and the microphone. Because this result stems from combined behavior of the microphone and the photoacoustic cell, it is necessary to analyze each component and unravel how they are coupled. In particular, we hypothesized that when the resonance peak of the photoacoustic cell overlaps with the resonance of the microphone, the largest signal amplitude can be obtained, as well as the highest Q-factor, sensitivity, and signal-to-noise ratio. As a result of matching the resonance of the photoacoustic cell with that of the microphone, the Q-factor increased from 11.8 (only with the microphone, Figure 2) to 21.0. The low frequency resonance exists near 16 kHz with the photoacoustic cell (see Figure 3b) but it is smaller than the value seen in Figure 2 with the microphone only. One possible explanation for this is that the first resonance of the microphone at 16 kHz was not as well coupled with the acoustic cell as the second resonance of the microphone at 47 kHz.

To test if the sensitivity is enhanced by matching the resonance of a photoacoustic cell with that of a microphone, we altered the geometry of the photoacoustic cell and measured the frequency response of the photoacoustic signal from 1 kHz to 80 kHz in a step of 0.1 kHz. The diagram of a photoacoustic cell in Figure 3a illustrates the dimensions for the following altered geometries of the photoacoustic cell. We changed the length of the branch cavity from 5 mm to 11 mm and the height and diameter of the main cavity from 7 mm to 10 mm and from 4.5 mm to 14.5 mm, respectively, as these parameters would affect the frequency response. While varying one dimension, we fixed the remaining dimensions at 8 mm, 8.5 mm, and 15 mm in length, diameter, and height, respectively, by default. Figure 3c–e present the frequency response of photoacoustic cells with each altered geometry between 35 kHz and 65 kHz to show clear differences between shifting resonance peaks. The longitudinal mode of the resonances in the branch cavity or the main cavity was believed to be one of the most important factors to find the most optimal point of operation. Significantly, we found that the effects of the branch cavity length and the main cavity height (Figure 3c,d) were negligible compared to the effect of the radial mode of the resonance in the main cavity (Figure 3e). The height of the main cavity had little impact on the frequency response as well. However, as shown in Figure 3e, the resonance peak shifted from left to right in the frequency response curves as the diameter decreases. Therefore, neither the resonance of the longitudinal mode in the branch cavity nor that in the main cavity is a significant factor to optimize the cell, but the radial mode of resonance mainly governs the acoustic characteristic of the photoacoustic cell. Importantly, we observed that the photoacoustic peak was separated from and merged with the microphone resonance peak (Figure 3e). In addition, the magnitude of the peak gradually increased up to 47 kHz as the resonance peak shifted and decreased thereafter. To determine if the microphone has a direct effect on the frequency response curve, we rescaled the microphone response curve of Figure 2 and overlapped it with the response of the photoacoustic cell of Figure 3e. Here, the microphone response enveloped the peaks of each frequency response of photoacoustic cells. The overlapped microphone curve has a slight difference in the resonance frequency from the 8.5 mm cavity’s resonance frequency. In this study, we focus on demonstrating the use of a microphone resonance with a resonance-matched cell in photoacoustic spectroscopy without additional minor tuning.

If the resonance of the branch cavity is not used, the signal presumably becomes smaller as the length increases. However, we observed that the signal did not monotonically decrease. Specifically, the signal decreased by 15% from a 5 mm long branch cavity to a 6 mm long branch cavity, increased from a 7 mm cavity to an 8 mm cavity, and decreased by 15% again from a 9 mm cavity to an 11 mm. The signal magnitude of the photoacoustic cell having the branch cavity of 8 mm in length was close to that of the photoacoustic cell having the branch cavity of 5 mm in length (<5% difference). The acoustic resonance thus relies on the geometries of both the main cavity and the branch cavity, despite the fact that the main cavity is responsible for the dominant effect. Nevertheless, compared to the significant changes in the signal when the main cavity diameter (>350%) changes, the signal change by varying the branch cavity length is relatively small. We here focus on demonstrating the microphone’s resonant behavior and optimization of the photoacoustic cell for the microphone’s resonance. Further investigation of the combined effect of the both cavities is planned in future studies, as previously reported by Schill et al. [24].

### 3.3. Analytical and Numerical Simulation of Photoacoustic-Cell Resonace

**Figure 4:**
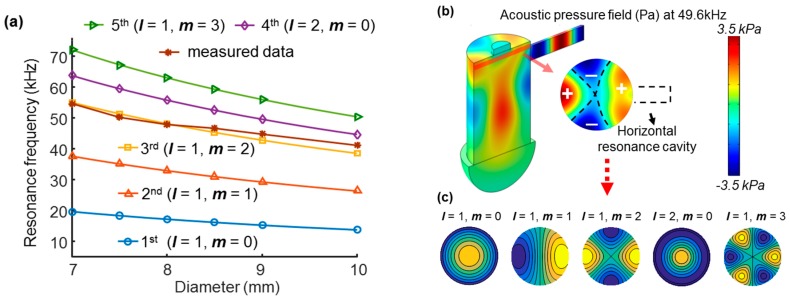
The third radial and azimuthal mode (*l* = 1, *m* = 2) of resonance is in accord with the experimental data. (**a**) Resonance frequencies calculated analytically for the first five modes of radial and azimuthal mode, compared to those obtained experimentally with varying diameter. (**b**) Finite element simulation at the eigenfrequency of 49.6 kHz of the default cavity dimension. The cross-sectional view of the acoustic pressure field shows a pattern of the third radial and azimuthal mode of resonance. (**c**) Ideal patterns of the first five modes of radial and azimuthal resonance.

To explain the frequency response of the altered cell geometries, we compared the analytically calculated eigenfrequencies with the measured data. Our T-shape cavity does not have a complete analytical solution for the acoustic resonance. However, because the length of the branch cavity had little impact on the frequency response, we first simplified our cell to be a simple cylindrical cavity without a branch. In addition, the diameter of the main cavity was a major factor to determine the resonance characteristics whereas the height of the main cavity had little impact; thus, we investigated the radial and azimuthal mode of resonance in the main cavity. Solving the eigenmode and eigenfrequency of the Helmholtz wave equation in a simple one-end open cylindrical cavity [25] gives the resonance frequency of radial and azimuthal modes of the following form:

(1) $$f(l,m)=\frac{c}{\pi }\frac{\alpha (l,m)}{D},$$

where ***c*** is the speed of sound, *α(l,m)* is a cylindrical cavity natural circular wavenumber, *D* is the diameter of the cylindrical cavity, and *l* and *m* are the mode number of radial and azimuthal resonance, respectively. The first five modes of radial and azimuthal resonance frequencies (no longitudinal mode, i.e., *n* = 0) are in the order of *α*(1,0), *α*(1,1), *α*(1,2), *α*(2,0), and *α*(1,3), which are 1.2556, 2.4048, 3.5180, 4.0793, and 5.5201. We calculated the resonance frequency of the first five modes when the diameter of the cylindrical cavity changes. Figure 4a shows the resonance frequency with varying diameter of the main cavity, which is calculated analytically assuming the speed of sound is 343 m/s at 20 °C. The resonance frequency extracted from the frequency response curves in our experimental data matched the third radial and azimuthal mode of the analytical solution (*l* = 1, *m* = 2). These results imply that the acoustic resonance in our cell is mainly due to the third radial and azimuthal mode.

To confirm the resonance mode in our T-cell, we further investigated the shape of the pressure distribution in the T-shape cavity at the eigenfrequency using a finite element simulation (COMSOL Multiphysics version 4.2, COMSOL Inc., Burlington, MA, USA). Figure 4b shows the total acoustic pressure distribution obtained from the finite element simulation. Here, we assumed the cavity-air interface as a sound hard wall leading to total reflection at the interface between air and stainless steel. For the opening of the main cavity we used a perfect impedance matched layer as an artificial absorbing layer simulating the open end of the cavity. The perfect impedance matched layer was a half-spherical shape located at the open end of the cavity (Figure 4b). The external source term generating the acoustic pressure source was a harmonically oscillating displacement of 0.01 mm, normal to the surface of the sample through the 2.5 mm diameter opening. The maximum mesh size was set to one-tenth of the speed of sound divided by the maximum modulation frequency of 100 kHz, resulting in an eigenfrequency of 49.6 kHz. The result remained consistent even when the size of the half-spherical dummy layer was enlarged. As shown in Figure 4b, the azimuthal symmetry is slightly degenerated due to the branch cavity. However, the cross-sectional view of the pressure distribution exhibited a clear azimuthal pattern. The branch cavity may selectively excite the third radial and azimuthal resonance mode by leading to azimuthal symmetry in that mode in the azimuthal direction, and consequently the other radial modes are less prominent than the third mode.

To compare the acoustic pressure distribution of the finite element simulation with that of the analytical solution, we consider the cross-sectional pressure distribution of the radial resonance modes predicted from the analytical solution in a simple cylindrical cavity. The solution of the Helmholtz equation at an acoustic resonance frequency in the radial mode results in an acoustic pressure distribution according to the following simplified relation, where the first order Bessel function is *J_m_*, the radial coordinate *r*, and the angular coordinate *θ* [25]:

(2) $$p_{l,m}(r,\theta )\propto J_{m}(r\alpha_{l,m}/R)\cos(m\theta -\phi_{m}).$$

Figure 4c shows the pressure patterns predicted from the first five radial and azimuthal resonance modes in a simple cylindrical cavity calculated by Equation (2) with zero phase, $\phi_{m}$ = 0 (Matlab 2016a, Mathworks, Inc., Natick, MA, USA). The acoustic pressure distribution shows that the existence of the branch cavity distorts the shape of resonance while the shape of the pressure distribution was close to the third radial and azimuthal mode (*l* = 1, *m* = 2). This confirms that the third radial and azimuthal mode of acoustic resonance explains the majority of the acoustics in the cell and is the dominant mode over other modes obscured in the measured frequency response curve. The resonance of the longitudinal modes in the main cavity and branch cavity were weak compared to a radial resonance mode because the cavities have an ‘open’ end, which leads to radiation loss. We previously reported that the resonance frequency shifted when we extended the height of the main cavity by relocating an inserted tube of 0.5 mm thickness [4]. However, as shown in Figure 3, the change in the diameter of 0.5 mm dramatically altered the frequency response whereas a change of height resulted in little difference. Putting these data together, the frequency shift with extended cavity height [4] is explained by the increasing effective diameter with the insertion of the tube, which is consistent with the present data.

### 3.4. Signal-to-Noise Ratio Enhancement by Matching the Microphone and Photoacoustic-Cell Resonance

**Figure 5:**
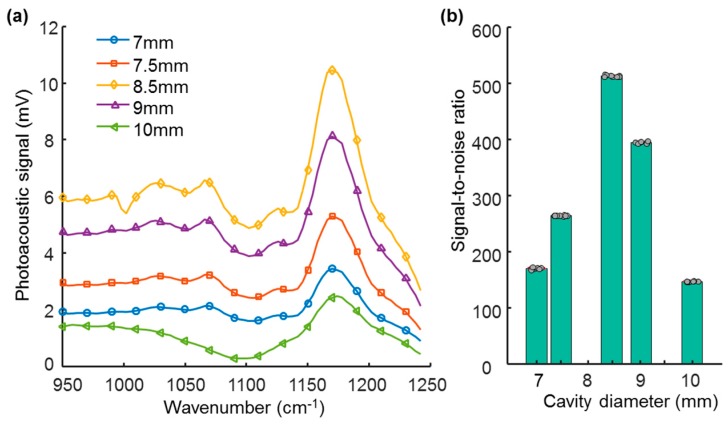
(**a**) Spectrum of carbon black sample from wavenumber scan when varying the diameter of main cavity and (**b**) signal-to-noise ratio depending on the cavity diameter. Each dot corresponds to the repeated acquisition of eight measurements.

We examined the signal-to-noise ratio enhancement of our photoacoustic spectroscopy system with an optimized photoacoustic cell by measuring the spectrum of a reference sample in photoacoustic cells of each altered diameter. Figure 5a shows the measured spectrum of the carbon black sample scanned over the wavenumber from 950 cm^−1^ to 1242 cm^−1^ in each photoacoustic cell while varying the diameter. The laser was modulated at the frequency where the largest peak of the frequency response was obtained. As expected, the cavity having an 8.5 mm diameter showed the largest photoacoustic signal. As shown in Figure 5b, the cell with an 8.5 mm diameter, where the acoustic resonance matches the microphone resonance, resulted in the largest signal-to-noise ratio (513, which is 3.5 times larger than that in the 10-mm diameter cavity).

## 4. Conclusions

In conclusion, by matching the resonance frequency of a photoacoustic cell with that of a microphone, we increased the sensitivity and signal-to-noise ratio of the photoacoustic spectroscopy system. The resonance mode was mainly in the radial and azimuthal direction, while the longitudinal mode had a negligible effect on the frequency response. These results provide guidance for those who develop photoacoustic spectroscopy using the ultrasound region and can be further applied to other ultrasound applications such as quartz-enhanced photoacoustic spectroscopy [26,27,28]. The method demonstrated here can be used to increase the signal-to-noise ratio and lower the detection limit of samples regardless of whether the sample is in open or closed form in all phases; liquid, solid, and gas. Similar T-cells were reported earlier where the branch cavity controls the acoustic resonance [23,29]. We employed a combined approach of analytical and numerical simulations and experimental analysis. The analysis of each characteristic of the microphone and photoacoustic cell can provide opportunities to tune the cell to the microphone resonance. This combinatorial approach can potentially be applied to mid-infrared photoacoustic spectroscopy for the non-invasive modality of glucose monitoring [1,2,4] to address the challenges of previously reported modalities.
